# Deep Synthesis of Realistic Medical Images: A Novel Tool in Clinical Research and Training

**DOI:** 10.3389/fninf.2018.00082

**Published:** 2018-11-20

**Authors:** Evgeniy Bart, Jay Hegdé

**Affiliations:** ^1^Palo Alto Research Center, Palo Alto, CA, United States; ^2^Department of Neuroscience and Regenerative Medicine, James and Jean Culver Vision Discovery Institute, The Graduate School, Augusta University, Augusta, GA, United States; ^3^Department of Ophthalmology, Medical College of Georgia, Augusta University, Augusta, GA, United States

**Keywords:** deep learning, deep neural network, perceptual metamerism, representational similarity analysis, signal detection, psychophysical

## Abstract

Making clinical decisions based on medical images is fundamentally an exercise in statistical decision-making. This is because in this case, the decision-maker must distinguish between image features that are clinically diagnostic (i.e., signal) from a large amount of non-diagnostic features. (i.e., noise). To perform this task, the decision-maker must have learned the underlying statistical distributions of the signal and noise to begin with. The same is true for machine learning algorithms that perform a given diagnostic task. In order to train and test human experts or expert machine systems in any diagnostic or analytical task, it is advisable to use large sets of images, so as to capture the underlying statistical distributions adequately. Large numbers of images are also useful in clinical and scientific research about the underlying diagnostic process, which remains poorly understood. Unfortunately, it is often difficult to obtain medical images of given specific descriptions in sufficiently large numbers. This represents a significant barrier to progress in the arenas of clinical care, education, and research. Here we describe a novel methodology that helps overcome this barrier. This method leverages the burgeoning technologies of deep learning (DL) and deep synthesis (DS) to synthesize medical images *de novo*. We provide a proof-of-principle of this approach using mammograms as an illustrative case. During the initial, prerequisite DL phase of the study, we trained a publicly available deep learning neural network (DNN), using open-sourced, radiologically vetted mammograms as labeled examples. During the subsequent DS phase of the study, the fully trained DNN was made to synthesize, *de novo*, images that capture the image statistics of a given input image. The resulting images indicated that our DNN was able to faithfully capture the image statistics of visually diverse sets of mammograms. We also briefly outline rigorous psychophysical testing methods to measure the extent to which synthesized mammography were sufficiently alike their original counterparts to human experts. These tests reveal that mammography experts fail to distinguish synthesized mammograms from their original counterparts at a statistically significant level, suggesting that the synthesized images were sufficiently realistic. Taken together, these results demonstrate that deep synthesis has the potential to be impactful in all fields in which medical images play a key role, most notably in radiology and pathology.

## Introduction

Medical images play an important role in many fields of modern medicine, and are outright central to some medical specialties, such as radiology and pathology. For instance, in screening mammography (Ritenour and Hendee, 1989; Sickles et al., 2002; Welch et al., 2016), the clinical decisions are primarily based on mammograms, and non-image information, such as the patient’s clinical charts, plays a supporting role: The radiologist must examine a given set of mammograms from a given patient along with other pertinent clinical information, and decide whether the patient must be recalled for additional tests. Given the enormous statistical variation among the images – after all, no two mammograms are exactly alike, even within a given patient – the mammographic examination amounts to a fundamentally image-based statistical decision-making task. That is, in order to make the correct decision, the radiologist must recognize very subtle, abstract image features that are diagnostic of an anomaly, and distinguish them from comparable features of a healthy breast. To recognize and utilize the diagnostic image features, the radiologist must be trained to do so in the first place. A large set of training images that reliably represent the diagnostic information, and the variability thereof, of real-world images would be needed to help provide the radiologist an adequate internal representation of the statistical distribution of the underlying diagnostic features.

The same is also true for training, testing and benchmarking machine learning applications designed to perform various analyses of medical images. Similar considerations also apply to clinical and scientific research involving analysis and perception of medical images (Samei and Elizabeth, 2010; Bhattacharyya et al., 2017; Marques et al., 2017). Altogether, for human experts and expert machines alike, the larger the training/testing medical image set, the better.

However, it can be quite difficult to get a sufficient number of images of a specific description. For instance, imagine training a radiology resident specializing in mammography. This is the field of medicine in which medical images are easiest to come by, in no small part because a relatively large proportion of eligible women typically undergo mammographic screening for breast cancer annually (Heath et al., 2001; Samei and Elizabeth, 2010). However, because the incidence of breast cancer is quite low (0.3–0.5% (Coldman and Phillips, 2013; Njor et al., 2013; Welch et al., 2016)), the proportion of images with cancer accounts for a tiny fraction of the available mammograms. Obviously, the incidence of each of the subtypes of breast cancer (e.g., ductal carcinoma *in situ*) will be even lower. Needless to say, in other fields of medicine where regular image-based health screening is less prevalent, available image sets tend to be even smaller.

Thus, it would be desirable to develop techniques for generating, *de novo*, a large number of medical images of a desired subtype. Here we demonstrate, using mammograms as a proof-of-principle example, that the machine learning method of deep learning (DL) followed by deep synthesis (DS) can be leveraged to help address this problem.

## Materials and Methods

### DL and DS: A Brief Background

In machine learning, DL generally refers to the use of deep (artificial) neural networks [DNNs; for reviews, see (Bianchini and Scarselli, 2014; LeCun et al., 2015; Kim et al., 2016; Aghdam and Elnaz, 2017; Hegdé, 2017)]. The underlying learning is referred to as “deep,” because DNNs typically consist of a hierarchy of many layers (dozens or even hundreds), all of which play a role in learning. This functional organization allows the network to learn a hierarchy of features of increasing complexity. The DNN must be adequately trained before DS can begin, so that the DL phase is a prerequisite for the DS phase.

During the ensuing DS phase, the fully trained DNN is made to synthesize one or more new images that capture the statistics of the training images. The specific method we used (Gatys et al., 2015, 2017; Wallis et al., 2017) further receives one additional “sample” image and synthesizes counterpart images, i.e., images that capture the statistics of the sample image specifically, rather than the general statistics of all images in the training set.

### Organization of the DNN

**FIGURE 1:**
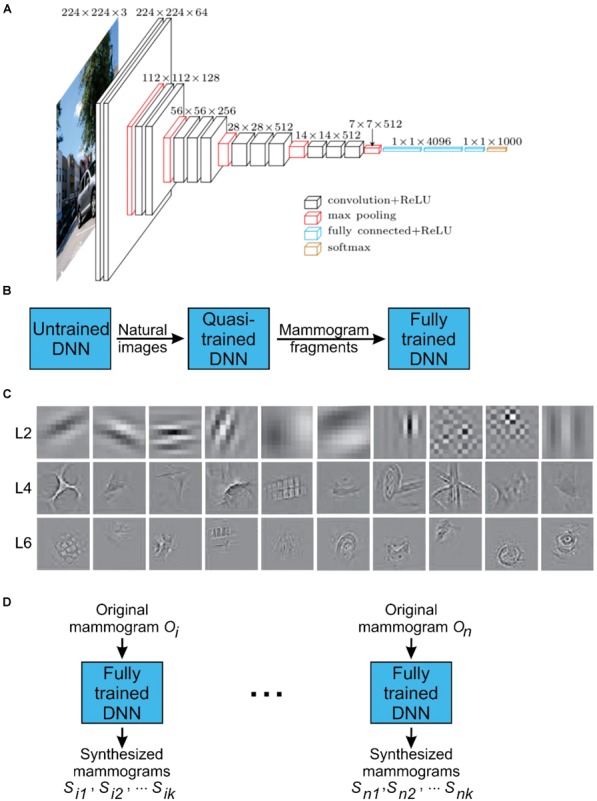
Configuration of the DNN used in this study. **(A)** A schematic representation of the DNN used for DL and DS of mammograms (Krizhevsky et al., 2012; Bart and Hegdé, 2018). Briefly, the network consists of 19 interconnected layers of various sizes (denoted by *stacked squares*) and a total of 650,000 neurons (not shown). **(B)** During the initial DL phase of the project, the DNN was trained with 1.2 million natural images. Subsequently, this quasi-trained network DNN was fine-tuned using about 9,000 partial view mammograms (PVMs) as training images generated from radiologically vetted actual mammograms as shown in Figure 2. This resulted in the fully trained network. This two-phase training process was necessary due to the relatively small number of mammograms available for training. **(C)** Receptive fields of selected neurons in layers 2, 4, and 6 in the fully trained network. **(D)** Deep synthesis of mammograms. For each input original mammogram *i*, the fully trained network can generate a virtually unlimited supply of synthesized mammograms.

In this study, we used a modified version of the publicly available DNN called VGGNet (Simonyan and Zisserman, 2015) (downloadable from http://www.robots.ox.ac.uk/~vgg/research/very_deep/). Briefly, the network consisted of 19 connected layers (Figure 1A). Neurons are heavily interconnected within layers, and less heavily across layers. The technical details of the construction, as well as the pre-training of this DNN using natural images, have been previously described in detail by Simonyan and Zisserman in (Simonyan and Zisserman, 2015)[also accessible at https://arxiv.org/abs/1409.1556].

Although the technical operational details of the DNN are rather involved, it is easy enough to develop an intuitive understanding of how it works. Briefly, as their name indicates, DNNs are organized as a network of neurons [for reviews, see (Cadieu et al., 2014; Cichy and Teng, 2017)]. The artificial neurons in question are idealized versions of biological neurons. Specifically, artificial neurons simulate the high-level computational properties of biological neurons, such as summing of the activations of input neurons (with appropriate synaptic weights) and firing (i.e., producing an output signal) when the sum exceeds a threshold. However, DNN neurons do not simulate the more detailed biological processes by which biological neurons perform the computations (such as membrane potentials, neurotransmitter release and re-uptake, etc.).

In the case of *image* processing DNNs, the neurons simulate the essential properties of the various levels of the primate *visual* system (Cichy et al., 2016, 2017). Thus, neurons in the input layers of the DNN simulate the retina, in that each one of them processes a small, localized region of the image that corresponds to the neuron’s receptive field. Neurons in each subsequent layer receive inputs from neurons in the previous layer, in a fashion broadly analogous to the actual brain. This allows the artificial network to construct a hierarchy of features of increasing complexity. Again, in analogy to biological visual systems, the receptive fields of neurons in the higher levels generally increase in size, so that neurons in the highest few layers can detect a feature anywhere in the visual field. The VGGNet is *convolutional* (Simonyan and Zisserman, 2015; Venkatesan and Baoxin, 2017; Chougrad et al., 2018), in that it replicates, with the same weights, each feature at every position in the input image. While generally not considered biologically plausible, this is a useful computational shortcut that allows significant reduction of both the computational requirements and the amount of necessary training data.

The deep learning neural network learns by adjusting the weights of the interconnections among the neurons based on the training images and the task. In general, the DNN does not retain every bit of information that is fed into it. Over the long run, it tends to learn only the statistical properties of the training images (e.g., in our case, what cancerous and healthy mammograms ‘look like’).

### DL Phase: Training and Fine-Tuning of the DNN Using Partial View Mammograms

The brand new, uninitialized DNN must be trained using a very large number (on the order of millions) of visual images. Since it was not possible to obtain this many mammograms, we used a DNN pre-trained using 1.2 million natural images as described in (Simonyan and Zisserman, 2015) (Figure 1B). This represents a workaround for the potential bootstrap problem, namely that synthesizing a large number of new medical images would require a large number of similar medical images to begin with. Obviously, this bootstrap problem applies to other types of medical images as well, and the above workaround is also likely to useful in such cases.

**FIGURE 2:**
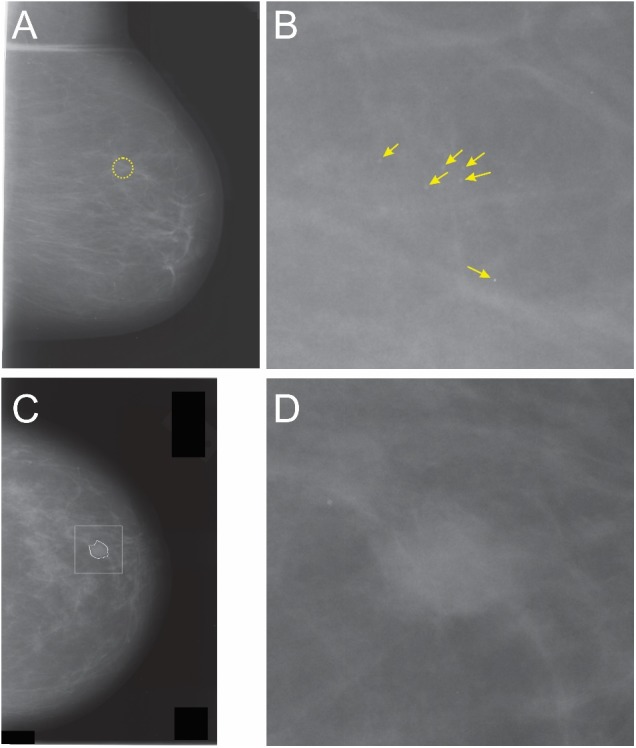
Whole-breast mammograms **(A**,**C)** and the corresponding partial view mammograms (PVMs) mammograms (**B**,**D**, respectively). The dotted circle in panel **A** denotes an ROI containing a cancer. Note that the cancerous region accounts for a relatively small portion (<1.4% of the area) of this mammogram, so that the rest of the image contains either healthy breast tissue, or a blank field that contains no breast tissue at all. Thus, diagnostic information in whole-breast mammogram is substantially “diluted.” Panel **B** shows a magnification-view mammogram of the same ROI that shows multiple microcalcifications (*arrows*). Note that this image is much enriched in diagnostic information compared to the whole-breast mammogram. It also has a much simpler global structure (or “image grammar,” in machine learning terms), in that it does not have the global information such as the breast outline, chest wall, musculature, etc. The same considerations apply to images in panels **C,D**. The irregular outline in panel **C** denotes the radiologically vetted ROI, and the square denotes the image region cropped from **C** to generate the mammogram fragment shown in **D**. See text for details.

After this initial training, the DNN was fine-tuned using about 9,000 mammogram images that we will refer to as “partial view mammograms” (PVMs). As their name indicates, PVMs are mammograms that focus on a small localized region of interest (ROI) of the breast, rather than the whole breast (Figure 2; see legend for details) (Kim et al., 2010). The rationale for using PVMs rather than whole-breast mammograms is threefold. First, radiologically vetted ROIs tend to account for a tiny fraction of the overall image area of a whole-breast mammogram (see, e.g., Figure 2). Thus, it is useful to ‘enrich’ the information content of training images by excluding the large remainder of the image that does not carry information of diagnostic interest. Second, the breast is a highly structured organ, and this structure is reflected in the image grammar of mammograms. The global structure of the breast (including the overall size, shape and outline) is far more variable than the local image features within the radiologically vetted ROIs, which would necessitate a far larger set of training images than are available publicly. Moreover, while capturing the image grammar is a higher-order problem of considerable future interest (see section “Discussion”), solving it is not necessary for demonstrating the applicability of the DL-DS methodology to medical images. Thus, using PVMs enabled us to focus on the first-order problem of adequately training the DNN by using the currently available image sets. Third, since mammogram fragments have far fewer pixels than whole breast mammograms, they can be handled by a relatively compact DNN, such as the one we used.

Partial view mammograms can be generated in one of two ways: They can be generated during diagnostic mammography (i.e., during mammographic imaging) intended to “zoom in” on an ROI, typically identified during the earlier screening mammography of the patient. A second way of generating PVMs is to generate mammogram fragments by digitally cropping a radiologically vetted, high-resolution ROI. In this study, we generated PVMs using the latter approach as described in greater detail below. It is important to note that both types of PVMs are used in various clinical contexts (Kim et al., 2010).

For this project, PVMs were generated from the whole-breast mammograms available in the Digital Database for Screening Mammography (DDSM) database (Heath et al., 2001; Venkatesan and Baoxin, 2017). Each mammogram belonged to one of the following four radiologically determined categories: “normal,” “benign without callback” (which were so obviously benign that the patient did not have to be re-examined), “benign” (ambiguous enough to necessitate patient callback, but eventually determined to be benign), and “cancer.”

For mammograms classified as “benign without callback,” “benign,” or “cancer,” the DDSM database also specifies radiologically determined outlines of the ROI (see, e.g., the *irregular white circle* in Figure 2C). To create the PVMs from such mammograms, we centered a 600 × 600 pixel square on the ROI (*white square* in Figure 2C). We skipped those mammograms for which any portion of the ROI lay outside of this square or the ones with more than one contiguous ROI. For the mammograms which had a single ROI that was fully contained in the aforementioned square, we generated the corresponding PVM by digitally cropping the square, so that one PVM was generated for each mammogram that met the aforementioned criterion.

For obvious reasons, the DDSM database defines no ROIs for normal mammograms. To generate “normal” PVMs, we identified the most visually salient region in each mammogram as described by Walther and Koch (Walther and Koch, 2006). We then centered the aforementioned 600 × 600 pixel square on the most salient pixel within the most salient region, and cropped the mammogram digitally to generate the corresponding PVM. This process generated roughly equal proportions of PVMs from each of the four classes of the mammograms.

Figure 1C illustrates some exemplar receptive fields of the fully trained DNN.

### DS Phase: *De novo* Synthesis of PVMs

The DS method we used is based on previous work by others (Gatys et al., 2015, 2017; Wallis et al., 2017). In this method, the fully trained DNN was presented with a series of additional, actual PVMs (“original mammograms”). For each original mammogram presented, the DNN was required to synthesize one or more images of the same size (“synthesized mammograms”) that captured the statistics of the original (see Figure 1D).

Briefly, DS is performed as follows. First, a feed-forward pass of the original mammogram through the DNN is performed. During this process, activations of all features in each layer are computed. Next, for each pair of features in a given layer, the correlations between their activations are computed and organized into a Gram matrix for that layer. Effectively, the set of Gram matrices for all layers of the DNN represents the structure of the input mammogram by capturing the high-level statistics of its overall appearance, but abstracting away details such as the position of each feature or the intensity of each pixel. Images with similar sets of Gram matrices will therefore contain broadly the same features (such as breast tissue structure or possible lesions), but will vary in their precise location, number, arrangement, and details of appearance.

**FIGURE 3:**
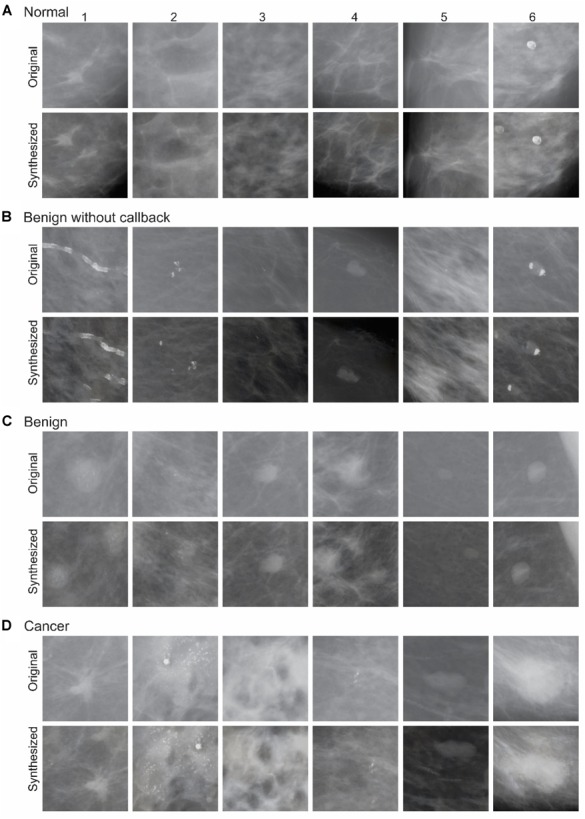
Exemplar images of the DS phase. During each round of synthesis, the fully trained network was provided *n* original mammograms as input, and was made to synthesize one or more output images. In this figure, selected input images from various diagnostic categories **(A–D)**, along with an exemplar synthesized counterparts, are shown. See text for details.

Next, one or more synthesized mammograms are generated that have Gram matrices similar to the original input mammogram. This is performed by first feeding a random white noise image through the DNN and computing the activations of all features. The Gram matrices for this synthetic image can then be computed. An objective function is then formulated as a simple L2 norm between the corresponding elements of the original and the synthesized Gram matrices. This objective function is optimized when the Gram matrices of the synthesized image are the same as the Gram matrices of the original image, i.e., when the synthesized image has similar features to the original. To synthesize a new mammogram, the objective function is optimized by gradient descent on the pixels of the image being synthesized. This process results in the generation of an image that has high-level features, properties and statistics similar to the original, but is different in the exact details (see Figure 3 for examples). Additional details can be found in (Gatys et al., 2015, 2017; Wallis et al., 2017). The relevant code is publicly available in (Gatys, 2015) (see also various code repositories referenced in (Gatys et al., 2015, 2017; Wallis et al., 2017)).

### Hardware Requirements

Training (and, to a lesser degree, using) DL methods is generally computationally expensive. For example, DL methods as yet cannot be used on mobile devices and require specialized hardware, most commonly GPUs. However, both training and using DL methods can be readily and efficiently carried out on hardware that is commercially and widely available, and is within reach for most research laboratories. The experiments described here were performed on a server with the following configuration:

- Two Intel Xeon E5-2650 V4 12 Core/24 thread CPUs @ 2.2 GHz Base/2.9 GHz Turbo.
- 512 GB RAM.
- Ten NVIDIA 1080 TI CPUs with 11 GB memory each.

While this configuration is decidedly more powerful and expensive than a typical workstation, the price tag of less than $30,000 makes it more affordable than most other pieces of equipment in a typical medical research laboratory. In addition, note that less expensive configurations would have worked as well. For example, the DS method we used ran on a single GPU using less than 1 GB of GPU memory and less than 1 GB of RAM. In this latter case, only one CPU thread was used, and only for control at that, rather than for actual computation.

### Human Psychophysical Testing

#### Subjects

All study subjects were practicing radiologists specializing in mammography who volunteered to participate in this study. All subjects gave written informed consent prior to participating in the study. All procedures related to study subjects were approved in advance by the Institutional Review Board (IRB) of Augusta University. This study was carried out in accordance with the enterprise human research protection policies and practices of the Augusta University Institutional Review Board. The underlying protocol was approved by the Augusta University Institutional Review Board. All subjects gave written informed consent in accordance with the Declaration of Helsinki.

All subjects were tested at the Perception Lab psychophysical testing facility organized by the National Cancer Institute at the 2017 annual meeting of the Radiological Society of North America (RSNA 2017) in Chicago, Ill. Each subject participated in one or both of the following two experiments, depending on his/her interest and availability. Only the data from those subjects who had at least ten trials for each image and tested in at least one experiment were further analyzed. Data from 17 subjects, corresponding to four original images and their four synthesized counterparts, met these criteria.

#### Stimuli

**FIGURE 4:**
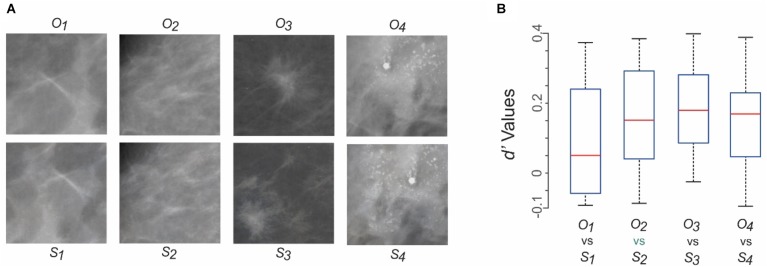
Can practicing radiologists tell the synthesized mammograms from their original counterparts? Results of Experiment 1. **(A)** Mammograms tested in Experiment 1. Four different original mammograms (*O_1_* through *O_4_*) and their synthesized counterparts (*S_1_* through *S_4_*) were chosen as test stimuli. Fourteen radiologists, each a practicing mammography specialist, viewed each image *ad libitum*, and reported whether it was a synthesized image or an original one. See text for details. The first two columns from left represent healthy breasts, and the last two columns represent cancerous ones. **(B)** The subjects’ responses were analyzed using *d’* analysis. The resulting *d’* values are shown as four box plots corresponding to the four pairs of stimuli in panel **A**. For each box plot, the top and bottom “*whiskers*” represent the maximum and minimum observed *d’* values, respectively. The *rectangle* denotes the interquartile range between the first and third quartile of observed values. The *red horizontal line* denotes the median value. None of the *d’* values were statistically significant (*p* > 0.05 for all values).

Subjects were tested using four pairs of original and synthesized images (two each from healthy and cancerous breasts; see Figure 4A). Note that we synthesized hundreds of images during the DS phase, 24 of which are shown in Figure 3. The rationale for testing a much smaller number of images during human testing is twofold: First, it is advisable to test each possible pair of images multiple times, so as to minimize the probability of Type II errors (i.e., of falsely concluding that the synthesized images are indistinguishable from original mammograms) due to low statistical power. On the other hand, using a larger number of images would result in a combinatorial explosion of the required number of trials, correspondingly reducing subject comfort. Second, as noted above, our primary goal was to establish that the DS/DL methodology is potentially feasible at least in some cases, and not that it is broadly applicable to all or most cases. Therefore, our approach represented practical balancing of all these considerations.

### Experiment 1: Perceptual Distinguishability of the Original vs. Synthesized Mammograms

All stimuli were presented against a neutral gray background. Each trial started with the presentation of a small (0.3°) central fixation spot on a neutral gray background. Subjects were told to fixate on the spot and indicate their readiness for the trial by pressing a key, upon which a single mammogram (16° × 16°) was presented. Subjects were informed that there was 50–50 chance that a given mammogram was original or synthesized. Subjects were told to scrutinize the mammogram *ad libitum* and report, using an appropriate key press, whether the mammogram was synthesized or not. The given trial ended with the subject’s response. Each mammogram was presented at least 10 times during randomly interleaved trials.

### Experiment 2: Representation Similarity Analysis (RSA) of the Mammograms

Experiment 2 was identical to Experiment 1 above, except as follows: During each trial, a pair of mammograms was presented side by side. Depending on the trial, either, both, or neither mammogram was an original mammogram. Subjects were told to scrutinize the mammograms *ad libitum* and report, using a mouse-driven on-screen slider, how diagnostically similar they were on a scale of 0 (identical) to 100 (nothing comparable). Each pair of mammograms was presented at least 10 times during randomly interleaved trials.

### Data Analysis

Data were analyzed using software custom-written in R (R Core Team, 2015) or Matlab (Natick, MA, United States). Representational Similarity Analyses [RSAs; (Shepard and Susan, 1970; Shepard et al., 1975; Kriegeskorte et al., 2008)] were carried out using Matlab scripts that utilized the RDM Toolbox (Nili et al., 2014) or custom-written software.

## Results

Figure 3 shows some examples of original mammograms used as input images during the DS phase, and the corresponding synthesized mammograms in each of the aforementioned four diagnostic categories. A few properties of the synthesized mammograms are particularly worth noting. First, by visual inspection, the synthesized mammograms appear quite similar in character to their original counterparts. Second, the synthesized mammograms are nonetheless pixel-wise quite different from their original counterparts. For instance, in the far left column of Figure 3, the bright spots indicating microcalcification are distributed differently in the synthesized mammograms compared to their original counterparts. This is also true of other input mammograms that are mutually quite different in physical appearance. Thus, the DNN appears to have learned some of the key statistical properties of the input mammograms.

These observations straightforwardly raise the question of whether the synthesized mammograms also appear perceptually indistinguishable, or “metameric” (Wyszecki and Stiles, 2000; Victor et al., 2015; Sweeny and Whitney, 2017), to trained experts. After all, it is a well-known property of visual pattern recognition expertise that experts can reliably distinguish abstract differences between visual objects that are too subtle for non-experts to recognize.

We tested this scenario using Experiment 1 (see section “Materials and Methods” for details). Figure 4A shows the four pairs of original and synthesized images used in this experiment. Fourteen practicing radiologists, each a practicing mammography specialist, viewed each image *ad libitum*, and reported whether it was original or synthesized. Each subject viewed each image ≥ 10 times in randomly interleaved trials (see section “Materials and Methods” for details).

We compared the nominal hit rate (i.e., proportion of trials in which the subject correctly identified a given image as synthesized) with the false alarm rate (i.e., proportion of trials in which the subject incorrectly identified a given image as synthesized) using the *d’* analysis. The resulting *d’* values showed that for each of the four pairs of images, no subject performed at a *d’* of > 0.4 (Figure 4B). That is, for each image pair, subjects failed to distinguish the synthesized image from its original counterpart significantly above chance levels (*p* > 0.05). Similar results were obtained (not shown) when we carried out a complementary *d’* analysis, in which we recalculated the *d’* values by comparing the nominal true negative rate (i.e., proportion of trials in which the subject correctly identified a given image as the original) with the misses (i.e., proportion of trials in which the subject incorrectly identified a given image as the original). Other response measures, such as the reaction time, also failed to show significant differences between synthesized and original images for any image pair (data not shown). Together, these results indicate that the synthesized images were perceptually metameric with their original counterparts, and vice versa.

The above results raise two interrelated questions. First, did the radiologists perceive the same diagnostic information in the synthesized image that they perceived in its in original counterpart? Second, was the perceived diagnostic information similar from one radiologist to the next?

Measuring internal perceptual representations of subjects is evidently extremely difficult. Fortunately, representational similarity analysis (RSA) provides a well-established, theoretically sound, quantitative approach to addressing these questions (Shepard and Susan, 1970; Edelman, 1998; Kriegeskorte et al., 2008; Cichy et al., 2014; Haxby et al., 2014; Guntupalli et al., 2016). We therefore carried out an RSA of diagnostic information in the aforementioned four image pairs (Experiment 2, see section “Materials and Methods” for details). In this experiment, twelve practicing mammography specialists, including nine subjects from Experiment 1, rated randomly drawn pairs of images from Figure 4A on a 0 – 100 scale as to how similar they were in terms of the diagnostic information they contained.

**FIGURE 5:**
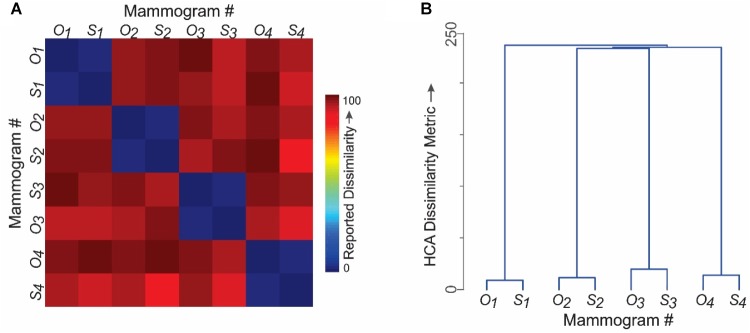
Do different radiologists perceive the same diagnostic features in synthesized mammograms? Results of Experiment 2. The four pairs of images shown in Figure 4A were used in this experiment to carry out a representational similarity analysis (RSA). Subjects rated the perceived similarity of diagnostic information between randomly selected pairs of mammograms on a 0–100 scale. See text for details. **(A)** Perceived pairwise similarities reported by fourteen practicing radiologists. Note that the corresponding pairs of original and synthesized counterparts were rated as highly similar (*blue hues*). **(B)** Hierarchical cluster analysis of the results in panel **A**. The vertical distance between any two mammograms is a quantitative measure of the distance between them in the perceptual space.

The mean reported similarities are shown as a conventional representational *dissimilarity* matrix (RDM) in Figure 5A in color-coded format. It is evident from the RDMs that the subjects tended to rate corresponding pairs of synthesized and original images as highly similar in terms of the diagnostic information they contained (*cells with blue hues* in Figure 5A). Conversely, subjects tended to rate unpaired images as containing highly dissimilar diagnostic information, regardless of whether the images were synthesized or original. Altogether, there were no significant differences between the similarity ratings elicited by original images *vs*. their synthesized counterparts (paired *t*-test, *p* > 0.05), thereby providing additional evidence that the two sets of images were perceptually indistinguishable. Moreover, the RDMs were statistically indistinguishable across subjects (RDM congruity analysis, *p* > 0.05; data not shown), suggesting that different subjects perceived the same underlying perceived diagnostic information.

The RDM data are shown as a hierarchical clustering analysis (HCA) plot in Figure 5B, where the vertical distance between any two images is a quantitative measure of the perceived similarity of the diagnostic information between them.

Together, these results indicate that practicing radiologists perceive similar diagnostic information in original images as they do in their synthesized counterparts.

## Discussion

Taken together, our results provide a proof of the principle that inter-related technologies of DL and DS can be utilized to synthesize medical images that are perceptually indistinguishable from their original counterparts even for expert observers. For reasons outlined in the Introduction, this is an important technical capability in the diverse fields of medical image evaluation, training, and research.

As detailed above, our DNN was implemented on common, commercially available GPU hardware, using publicly available software (Krizhevsky et al., 2012). Together, these considerations show that DS is a potentially powerful, versatile approach for overcoming one of the most important barriers to progress in clinical care and research in image-intensive fields of medicine.

### Some Important Caveats

We hasten to note some important caveats about the implications of our results. First, our results demonstrate only that our DNN *can* generate at least some realistic mammograms by objective criteria, and not that all mammograms generated by our DNN meet these criteria. This is important, because for practical reasons outlined in Materials and Methods, we were able to test only four, arbitrarily selected pairs of images. This constitutes only a small proportion of the images we generated using DS, which in turn represented but one category of medical images. This is why we emphasize that our study merely represents a proof of principle, and not the state-of-the-art. A second, related caveat is that the present study focused on synthesizing PVMs, and does not specifically address the applicability of this methodology to images with more complex grammars. On the other hand, there is no *a priori* reason to doubt that these methodologies would be applicable to all medical images, including those with complex grammars (see next paragraph). That is, the main goal of this report is to illustrate the potential of DL and DS to medical images. Therefore, it goes without saying that the approach outlined in this report has room for many tweaks and improvements, which we will briefly outline below.

### Future Directions

In contemplating the future applications of the DL/DS methodology to medical images, it is important to bear in mind that the entire field of DL, and especially DS, are both in their infancy. A number of remarkable, real-world applications of this technology serve to highlight the enormous future potential of this technology (LeCun et al., 2015; Hegdé, 2017). Therefore, any of the future directions noted below may fall squarely within the realm of feasibility within the next few years, if it is not there already.

#### Extending to Whole-Breast Mammograms

As noted above, whole-breast image statistics are far more variable that the statistics of the mammogram fragments used in this report. Given the relative scarcity of available training images (thousands rather than millions of mammograms are readily available), extending our results to whole-breast mammograms will require some novel approaches to learning the appropriate image grammar.

#### Extending to Other Types of Medical Images and Videos

Many other types of medical images of highly structured body parts, such as the lungs, the brain, the liver, and the prostate, would similarly require learning complex image grammars. In each of those cases, it may be possible to replicate our approach, whereby initial studies use image fragments to synthesize diagnostically informative image fragments before attempting to synthesize the whole-organ images.

It should be noted that some types of medical images, especially in pathology, have much simpler image grammars. For instance, pap smears contain an array of individual cells that tend to be arranged randomly with respect to each other (Cervical cancer screening: the Pap smear, 1980; Richart, 1980; Shield et al., 1987), so that the relative spatial configuration of the cells contains no diagnostic information. It should be much easier to extend our results to such fields, provided a requisite number of training images, or image fragments (e.g., fragments limited to individual cells), can be acquired.

Many fields of medicine, such as surgery, angiography, cardiology, colonoscopy, etc., rely more on three-dimensional (3D) video feeds (i.e., 4-D images, where the fourth dimension is time) as much as, or more than, static 2D images. It should be possible, in principle, to extend the DL/DS methodology to 4D images as well.

#### Systematically Manipulating Medical Images

As can be seen by comparing the original and synthesized counterparts in this report (Figures 3, 4A), our current methodology is capable of randomly manipulating medical images, e.g., by changing the image location of calcification. However, it would be even more useful to *systematically* manipulate information, e.g., systematically changing the location of the calcification.

#### Enhancing the Explainability of DL and DS to Human Users

So far, our collective understanding *what* DNNs can do has more than outpaced our understanding of *how* DNNs do what they do. That is, the operation of DNNs can be so abstract and opaque that it can hard to interpret or explain the outputs of the DNNs in understandable terms. Naturally, lack of explainability can reduce the confidence of the end users, or “clients”, in the computational output of expert machines.

While the explainability gap decidedly applies to our DNN, it is no means limited to it. Explainability is a common problem of DNNs in general, and of intelligent machines at large (de Visser, 2012; Gunning, 2016; Ribeiro et al., 2016; Doshi-Velez and Been, 2017; Holzinger et al., 2017).

Explainability of medical image synthesis is particularly important because the end users benefit by understanding how the methodology works, and the methodology benefits by trust and confidence that explainable system tend to inspire in end users (Elmore et al., 1994; Goldstein et al., 2015; Ribeiro et al., 2016; Fernandes et al., 2017; Holzinger et al., 2017; Hegdé, 2018).

#### Enhancing the Usefulness of DNNs With “Human-in-the-Loop”

As noted above, one of the main advantages of the DL/DS methodology in the context of medical images is that it can help overcome the barrier posed by limited availability of data sets. Machine systems can be made even more effective in overcoming the limitations of input data, or their own operational limitations, using human experts (Holzinger, 2016). This allows the biological expert “system” called the highly trained human mind to compensate for the shortcomings of the machine expert system, and vice versa. Including a human in the loop in this fashion is especially important in the context of medical images, both not only because the clinical decisions will ultimately have to be made by a human decision-maker, but also because the factors that hamper machine systems, such as the limitations of data, are an ever-present problem in medicine.

## Conclusion

Our results demonstrate that DS is a feasible approach for synthesizing medical images. With the aforementioned improvements, it is likely to be a powerful tool in clinical training, development of machine systems for clinical data analysis and decision-making, and clinical research.

## Author Contributions

EB and JH designed the study together and wrote the manuscript jointly. EB constructed and trained the network in consultation with JH, and generated new images. JH carried out the psychophysical experiments, and collected and analyzed the data in consultation with EB.

## Conflict of Interest Statement

The authors declare that the research was conducted in the absence of any commercial or financial relationships that could be construed as a potential conflict of interest.
